# β-glucan protects against necrotizing enterocolitis in mice by inhibiting intestinal inflammation, improving the gut barrier, and modulating gut microbiota

**DOI:** 10.1186/s12967-022-03866-x

**Published:** 2023-01-10

**Authors:** Xingdao Zhang, Yuni Zhang, Yu He, Xingwang Zhu, Qing Ai, Yuan Shi

**Affiliations:** 1 Department of Neonatology, National Clinical Research Center for Child Health and Disorders, Ministry of Education Key Laboratory of Child Development and Disorders, Children’s Hospital of Chongqing Medical University, Chongqing, China; 2 Chongqing Key Laboratory of Pediatrics, Chongqing, China

**Keywords:** β-glucan, Inflammatory cytokines, Tight junctions, Intestinal microbiota, TLR4-NF-κB

## Abstract

**Background:**

Necrotizing enterocolitis (NEC) is a devastating gastrointestinal disease with high morbidity and mortality, affecting preterm infants especially those with very low and extremely low birth weight. β-glucan has manifested multiple biological effects including anti-inflammatory, regulation of gut microbiota, and immunomodulatory activities. This study aimed to investigate the effects of β-glucan on NEC.

**Methods:**

Neonatal C57BL/6 mice were randomly divided into three groups: Control group, NEC group and β-glucan group. Newborn 3-day-old mice were gavaged with either 1 mg/ml β-glucan or phosphate buffer saline at 0.03 ml/g for 7 consecutive days before NEC induction and a NEC model was established with hypoxia combined with cold exposure and formula feeding. All the pups were killed after 72-h modeling. Hematoxylin–eosin staining was performed to assess the pathological injury to the intestines. The mRNA expression levels of inflammatory factors in intestinal tissues were determined using quantitative real-time PCR. The protein levels of TLR4, NF-κB and tight junction proteins in intestinal tissues were evaluated using western blotting and immunohistochemistry. 16S rRNA sequencing was performed to determine the structure of the gut microbiota.

**Results:**

β-glucan administration ameliorated intestinal injury of NEC mice; reduced the intestinal expression of TLR4, NF-κB, IL-1β, IL-6, and TNF-α; increased the intestinal expression of IL-10; and improved the expression of ZO-1, Occludin and Claudin-1 within the intestinal barrier. Pre-treatment with β-glucan also increased the proportion of *Actinobacteria*, *Clostridium butyricum, Lactobacillus johnsonii*, *Lactobacillus murinus*, and *Lachnospiraceae bacterium mt14* and reduced the proportion of *Klebsiella oxytoca g Klebsiella* in the NEC model.

**Conclusion:**

β-glucan intervention prevents against NEC in neonatal mice, possibly by suppressing the TLR4-NF-κB signaling pathway, improving intestinal barrier function, and partially regulating intestinal microbiota.

**Supplementary Information:**

The online version contains supplementary material available at 10.1186/s12967-022-03866-x.

## Introduction

Necrotizing enterocolitis (NEC) is a devastating gastrointestinal disease with high morbidity and mortality, affecting preterm infants, especially those with very low and extremely low birth weight [1, 2]. NEC is characterized by acute intestinal ischemia and necrosis and a mortality of approximately 25% in most cases; however, in the most severe cases, it can reach as high as 80% in 48 h after diagnosis [3]. At present, therapies for NEC are limited to cessation of feeding, hemodynamic resuscitation and the administration of broad-spectrum antibiotics [4]. However, for infants who continue to deteriorate rapidly, urgent surgery is required to excise a necrotic bowel [5]. Moreover, in recent years, the complications of NEC beyond the intestine including the lung and the brain have been noticed in NEC survivors [6, 7].

Although the definite pathogenesis of NEC remains not fully understood [8], it is generally acknowledged that the lipopolysaccharide receptor toll-like receptor 4 (TLR4) plays a key role in triggering mucosal inflammation. Microbial dysbiosis can activate TLR4 and ultimately leads to NEC [9, 10]. Exaggerated TLR4 signaling triggers a typical cascade that activates nuclear factor-κB (NF-κB) and induces the accumulation of proinflammatory cytokines [11]. The progression of the inflammatory cascade above leads to intestinal epithelial cell death by apoptosis and necroptosis, weakened mucosal reconstruction, and gut barrier disruption. The subsequently increased permeability, allows more toxic substances and pathogenic microorganisms to translocate into the underlying vascular network, ultimately resulting in progression to severe NEC [12]. Currently, there is no effective treatment for NEC. Preventive strategies to reduce the severity of the disease and elucidating underlying processes that lead to the long- term complications of NEC have been gradually emphasized [13].

β-glucan is a kind of bioactive polysaccharide obtained from yeast, mushrooms, algae, and cereals, such as oat β-glucan and *Lentinus edodes* β-glucan, which is believed to be beneficial for health and is edible to be taken orally as food supplements and daily diet [14–17]. It consists of β-D-glucose monomeric units linked together by glycosidic bonds at different positions, e.g., (1,3), (1,4), or (1,6). β-glucans and β-1, 3-D-glucans as well as β-1, 6-D-glucans derived from higher fungi, mushrooms, molds, and yeasts differ from β-1, 3-D-glucans and β-1, 4-D-glucans, which are primarily obtained from the cell walls and the seeds of some cereals [18]. Currently, β-glucan has demonstrated multiple biological effects including anti-tumor [19], anti-obesity [20], anti-allergy [21], anti-osteoporosis [22], anti-inflammatory [23, 24], regulation of gut microbiota [25–27], and immunomodulatory activities [28, 29]. Among them, fungal β-glucans have more significant immunomodulatory effects [30]. It has been reported that fungal β-glucan inhibited the release of lipopolysaccharide (LPS)-induced nitric oxide and tumor necrosis factor (TNF)-α in vitro and reduced the secretion of TNF-α and interleukin (IL)-6 in vivo [31, 32]. In a previous study, for the first time, Vetvicka et al. reported that supplementation of β-glucan reduced the incidence and severity of NEC in a neonatal rat model via reduction of inflammatory response within the intestine [33]. However, the precise mechanism by which β-glucans inhibit the release of inflammatory cytokines and induce anti-inflammatory immune cells is complex and not fully understood. It was previously reported that monocytes isolated from β-glucan-treated mice released fewer TNF-α and IL-6 after stimulation [34]. Another report has shown that β-(1,3)/(1,6)-glucan ameliorates mastitis by reducing proinflammatory cytokines TNF-α and IL-1β via the TLR4-MyD88-NF-κB signaling pathway [35, 36]. What’s more, a study has showed that *Lentinus edodes* β-glucan supplement could help attenuate DSS-induced colitis via MAPK-Elk-1 and MAPK-PPARγ pathways [37]. To investigate the preventive effects of β-glucan against NEC, we hypothesized that oral β-glucan supplementation could inhibit intestinal inflammation, improve the intestinal barrier and modulate the gut microbiota to protect against NEC by inhibiting the TLR4-NF-κB signaling pathway. To elucidate the preventive effect of β-glucan on NEC, we established a neonatal mouse model of NEC and gavaged with β-glucan before the establishment of NEC.

## Materials and methods

### β-glucan and its preparation

**Fig. 1:**
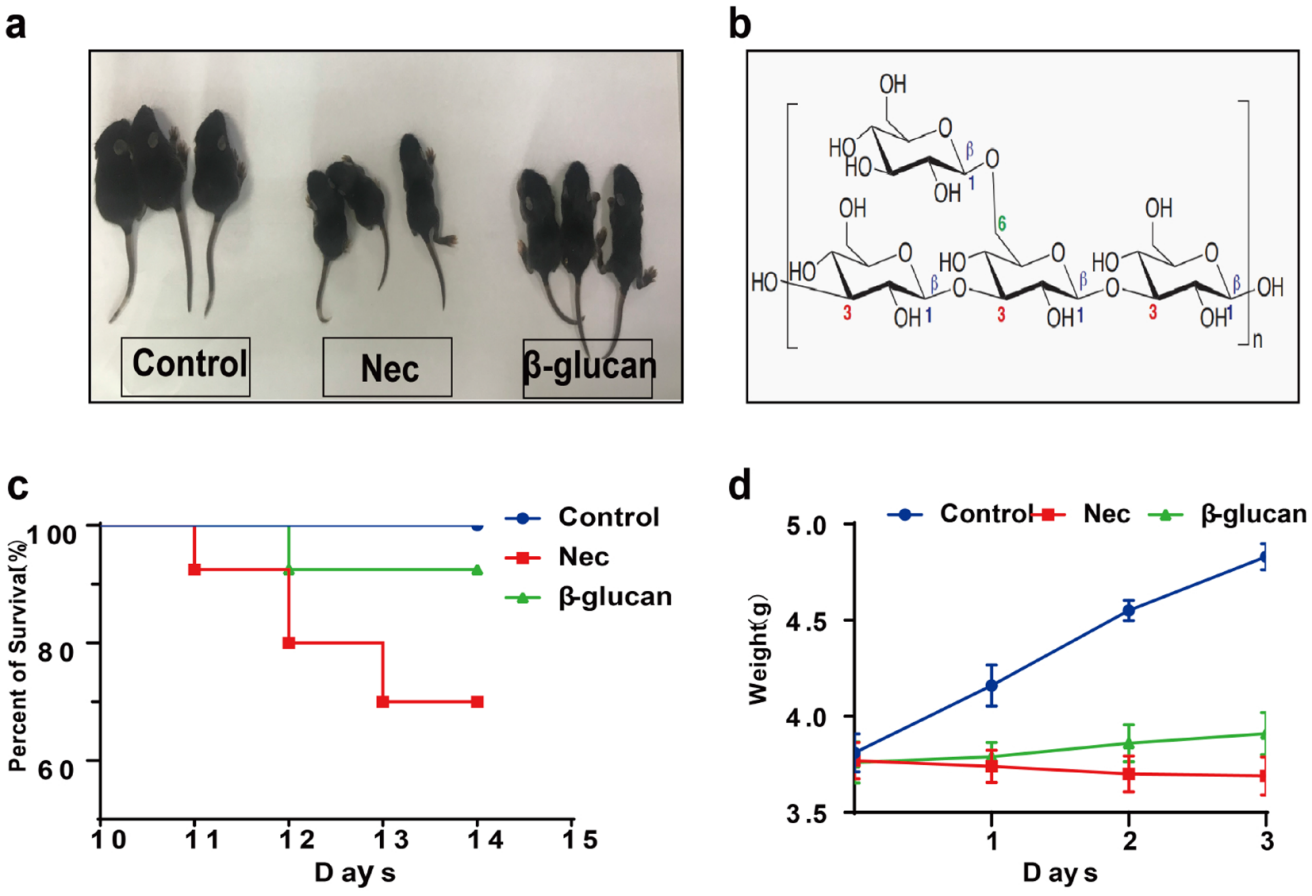
Changes in general conditions, body weight, and survival rate after the administration of β-glucan in mice with NEC. **a** Comparison of appearance among the three groups. **b** The main structure of β-glucan. **c** Survival curves of neonatal mice in the three groups. *n* = 40 **d** Body weight changes of neonatal mice in the three groups. *n* = 10

The β-glucan used in this study was β-glucan peptide, a high-molecular-weight polysaccharide extracted from the fungus *Trametes versicolor* (Invivogen, category code: tlrl-bgp). The β-glucan consists of a highly ramified glucan portion, including a β-(1,4) main chain and β-(1,3) side chain, with β-(1,6) side chains covalently linked to a polypeptide portion rich in aspartic, glutamic, and other amino acids. The main structure of β-glucan is shown in Fig. 1b. β-glucan was diluted in sterile phosphate buffer saline (PBS) to a concentration of 1 mg/ml.

### NEC induction and drug treatment

All operations performed in our experiment were approved by the Animal Ethics Committee at Chongqing Medical University. Following the protocol previously described [38], 10-day-old C57BL/6 mice were separated from their mothers and were fed by gavage with hyperosmolar formula (Similac Advance (Abbott Nutrition, USA)/Esbilac puppy milk replacer (PetAg, USA) = 1.7) every 4 h, subjected to hypoxia and hypothermia (100% N_2_ for 90 s subsequently at 4 ℃ for 10 min, 3 times per day) for 3 days to induce NEC. In our study, newborn 3-day-old mice were gavaged with either 1 mg/ml β-glucan or PBS at 0.03 ml/g for 7 consecutive days before NEC induction. In addition, age-matched and untreated mice were left with their mothers as the Control group. Body weight and survival condition were recorded daily throughout the establishment of NEC. On postnatal day 13, mice were sacrificed by cervical decapitation and the intestines were harvested for further analysis.

### Gut histology

The intestines were completely removed, and a 1-cm portion of the distal ileum was fixed in 4% paraformaldehyde solution overnight. Then, the samples were dehydrated, embedded in paraffin, and cut into 4-μm sections. Subsequently, 4-μm tissue sections were stained with hematoxylin and eosin, and an established scoring criterion [39] was used to perform the pathological injury score by two individual blinded pathologists (*n* = 6 pups/group). The details are as follows: 0 (normal): no damage; 1 (mild): slight submucosal and/or lamina propria separation; 2 (moderate): moderate separation of submucosa and/or lamina propria, and/or edema in submucosal and muscular layers; 3 (severe): severe separation of submucosa and/or lamina propria, and/or severe edema in submucosa and muscular layers, region villous sloughing; 4 (necrosis): loss of villi and necrosis. Mice with a pathological injury score more than 2 were considered NEC.

### Immunohistochemistry

Paraffin slices from the three groups (*n* = 4–6 pups/group) were deparaffinized in xylene and then in decreasing concentrations of ethanol (100% × 2 for 5 min, 85% for 5 min, and 75% for 5 min), followed by antigen retrieval with citric acid antigen repair buffer (pH 6.0) in a microwave oven. Endogenous peroxidases were blocked with 3% hydrogen peroxide, and then slices were covered with 3% bovine serum albumin for 30 min to block nonspecific binding. The slices were placed in a wet box and incubated with primary anti-TLR4, anti-ZO-1, anti-Occludin antibodies (Servicebio, China), and anti-NF-κB (Introvigen, US) diluted in PBS (pH 7.4) at 4 °C overnight. Slides were then washed in PBS three times for 5 min. After being slightly shaken dry, the slices were incubated with corresponding secondary antibody (HRP labeled) at room temperature for 50 min. Finally, slices were stained with DAB, and nuclei were stained using hematoxylin for 1 min. Images were collected using a slide scanner after dehydration and sealing.

### Real-time PCR

Total RNA was extracted from the intestine tissue using TRIzol (Life Technologies CA, USA) (*n* = 6–8 pups/group). The purity of RNA was quantified using a nanodrop spectrophotometer (Thermo Fisher Scientific, CA, USA) and eligible RNA samples (OD260/280 = 1.8–2.2, OD260/230 ≥ 2.0) were used. cDNAs were synthesized using a Prime Script RT Reagent (Takara, Japan) and were used for qRT-PCR assay using TB Green Premix Ex Taq II (Tli RNase H Plus) Kit (Takara, Japan). β-Actin was used as an internal control, and the relative expression of mRNA (TLR4, IL-1β, IL-6, IL-10, and TNF-α) in intestine tissue was determined using the ΔΔCT method. Detailed information on the RT-PCR primer sequences is shown in Additional file 1: Table S1.

### Western blotting

Intestine tissue (*n* = 4 pups/group) soaked in RIPA lysis buffer (Beyotime, China) supplemented with protease inhibitor (Beyotime, China) was homogenized using an electric homogenizer and centrifuged to obtain the supernatant. Protein concentrations were measured using a Pierce BCA Protein Assay Kit (Beyotime, China). The protein supernatant was mixed with sodium dodecyl sulfate sample buffer (Beyotime, China) at a ratio of 4:1 and denatured at 100 ℃ for 10 min. Protein samples were separated in 10% polyacrylamide gels and transferred to 0.45 μm PVDF membranes, and measured using anti-TLR4, anti-NF-κB P65 (Servicebio, China), anti-ZO-1, anti-Occludin, anti-Claudin-1 (Proteintech, China) and β-Actin (ZENBIO Biotechnology, China) at 4 ℃ overnight. Signals were detected using chemiluminescence (ECL Western Blotting Substrate, Bio-Rad). The relative intensity of target bands was quantified using the Image J analysis system (Bio-Rad).

### Fecal sample collection and microbiota analysis

The ileum and colon feces of mice were collected with sterile ophthalmic forceps and placed in 1.5 ml sterile tubes immediately. Then the samples were put into the liquid nitrogen and transferred to a – 80 ℃ refrigerator for fecal microbiota analysis (*n* = 7 pups/group). Total genomic DNA was extracted from fecal samples using the E.Z.N.A.^®^ stool DNA Kit (Omega Bio-Tek, Norcross, GA, USA) according to the manufacturer’s instructions. The DNA quality and concentration were monitored using 1% agarose gel electrophoresis and a NanoDrop^®^ ND-2000 spectrophotometer (Thermo Scientific Inc., USA). All the procedures were conducted in clean bench and all the equipment used in thees operations were sterilized by autoclave. The V3-V4 region of bacterial 16S rRNA genes was amplified using the 338F-806R primer pairs: 338F (5'-ACTCCTACGGGAGGCAGCAG-3') and 806R (5'-GGACTACHVGGGTWTCTAAT-3') in an ABI GeneAmp^®^ 9700 PCR thermocycler (ABI, CA, USA). PCR amplification cycling conditions were as follows: initial denaturation at 95 ℃ (3 min), 27 cycles of denaturing at 95 ℃ (30 s), annealing at 55 ℃ (30 s) and extension at 72 ℃ (45 s), single extension at 72 ℃ (10 min), and end at 4 ℃. All samples were amplified in triplicate, in parallel with a negative control (DNase-RNase-free water). The PCR product was extracted from 2% agarose gel after electrophoresis detection, purified using the AxyPrep DNA Gel Extraction Kit (Axygen Biosciences, Union City, CA, USA) according to the manufacturer’s instructions and quantified using a Quantus^™^ Fluorometer (Promega, USA). Purified amplicons were pooled in equimolar amounts and paired-end sequenced on an Illumina MiSeq PE300 platform (Illumina, San Diego, USA). The raw data were uploaded in the NCBI Sequence Read Archive (SRA) database (BioProject ID: PRJNA906728 http://www.ncbi.nlm.nih.gov/bioproject/906728). The raw FASTQ data were quality-filtered by FASTP version 0.19.6 and then merged by FLASH version 1.2.7 with the following criteria: reads that contain ambiguous characters or could not be assembled were discarded; reads with a quality score of less than 20 were truncated; low complicity reads were removed; and paired sequences were merged with an overlapped length greater than 10 bp. The maximum mismatch ratio of the overlapping region is 0.2. Using UPARSE 7.1, the optimized sequences were clustered into operational taxonomic units (OTUs) based on 97% sequence similarity. The most abundant sequence was selected as a representative sequence from each OTU. To minimize the effects of sequencing depth on alpha and beta diversity measures, the number of 16S rRNA gene sequences from each sample was rarefied to 27,555. Bioinformatic analysis of the gut microbiota was conducted using the Majorbio Cloud platform (https://cloud.majorbio.com). Based on the information on OTUs, rarefaction curves and alpha diversity indices including observed OTUs, Chao1 richness, Shannon index, and Good’s coverage were calculated using Mothur v1.30.1. The similarity among the microbial communities in different samples was determined using principal coordinate analysis (PCoA) based on Bray Curtis dissimilarity. The PERMANOVA test was performed to assess the percentage of variation explained by the treatment along with its statistical significance (*n* = 7). The linear discriminant analysis (LDA) effect size (LEfSe) was used to identify the significantly abundant taxa (phylum to genera) of bacteria among the three groups (LDA score > 2, *P* < 0.05).

### Molecular docking

Molecular docking was used to identify the binding mode between the β-glucan and the TLR4 using AutoDock4.2. The structure of TLR4 (PDB ID: 3FXI) was downloaded from Research Collaboratory for Structural Bioinformatics Protein Data Bank [40]. (RCSB PDB, RRID:SCR_012820) (http://www.rcsb.org/pdb/). The 3-D structure of the β-glucan peptide was drawn using RDKit. The protein Amber14SB charge and the protonation state were allocated using UCSF Chimera software and H +  + , respectively [41, 42], and the structure was optimized using the classical MMFF94 force field. The optimized molecules were employed for an AM1-BCC local charge calculation with UCSF Chimera software. The geometric center of the binding site that was predicted by SiteMap was applied as the docking center. The docking center of the TLR4 was identified as center_x: − 7.88, center_y: − 13.00, and center_z: 45.55. The docking calculation was limited to the rectangular box with the center of each protein docking, the side length was 22.5 Å, and the Spacing step was set to 0.375 Å. The maximum number of search conformations was set to 10,000. Amino acids in the docking center as well as ligands were regarded as flexible objects, and the outside amino acids were regarded as rigid objects, allowing amino acid side chains, such as aspartic acid and tryptophan, to flip over. A semi-flexible docking method was carried out for docking and a genetic algorithm was used for conformational sampling and scoring.

### Statistical analysis

Data analysis was performed using the GraphPad Prism (version 9.3.0). Normally distributed data were expressed as the mean ± SD, and significance was identified using one-way ANOVA. Median and interquartile range were used to describe nonnormally distributed data, and differences were determined using the Kruskal–Wallis test. *P* < 0.05 was considered statistically significant.

## Results

### β-glucan efficiently ameliorated intestinal injury in mice with NEC

#### Changes in general conditions, body weight and survival rate

General conditions such as vitality, weight gain, hair luster, and subcutaneous fat showed no obvious differences among the three groups before modeling. However, mice in the NEC group began to develop abdominal distension, low vitality, obvious weight loss, diarrhea, and hematochezia during modeling, whereas the β-glucan group showed abdominal distension, decreased activity but without diarrhea and hematochezia (Fig. 1a). The body weight of the NEC group mice decreased more than that of the β-glucan group mice on the 2nd day of modeling (*P* < 0.0001). Before sacrifice, body weight was significantly higher in the β-glucan group than in the NEC group (Fig. 1d,  P< 0.0001). No deaths were observed in the three groups before modeling. As shown in Fig. 1c, we did not observe deaths in the Control group during the 3 days of modeling. In the NEC group, three deaths occurred on the 1st day, five deaths occurred on the 2nd and four on the 3rd days. In the β-glucan group, three deaths occurred on the 2nd day, and zero on the 3rd day. The final survival rate differs significantly among the three groups: 70% (28/40) in the NEC group, 92.5% (37/40) in the β-glucan group, and 100% in the Control group (*P* < 0.001).

#### Effects of β-glucan on gross morphology, pathological morphology, and histological scoring of intestinal tissue

**Fig. 2:**
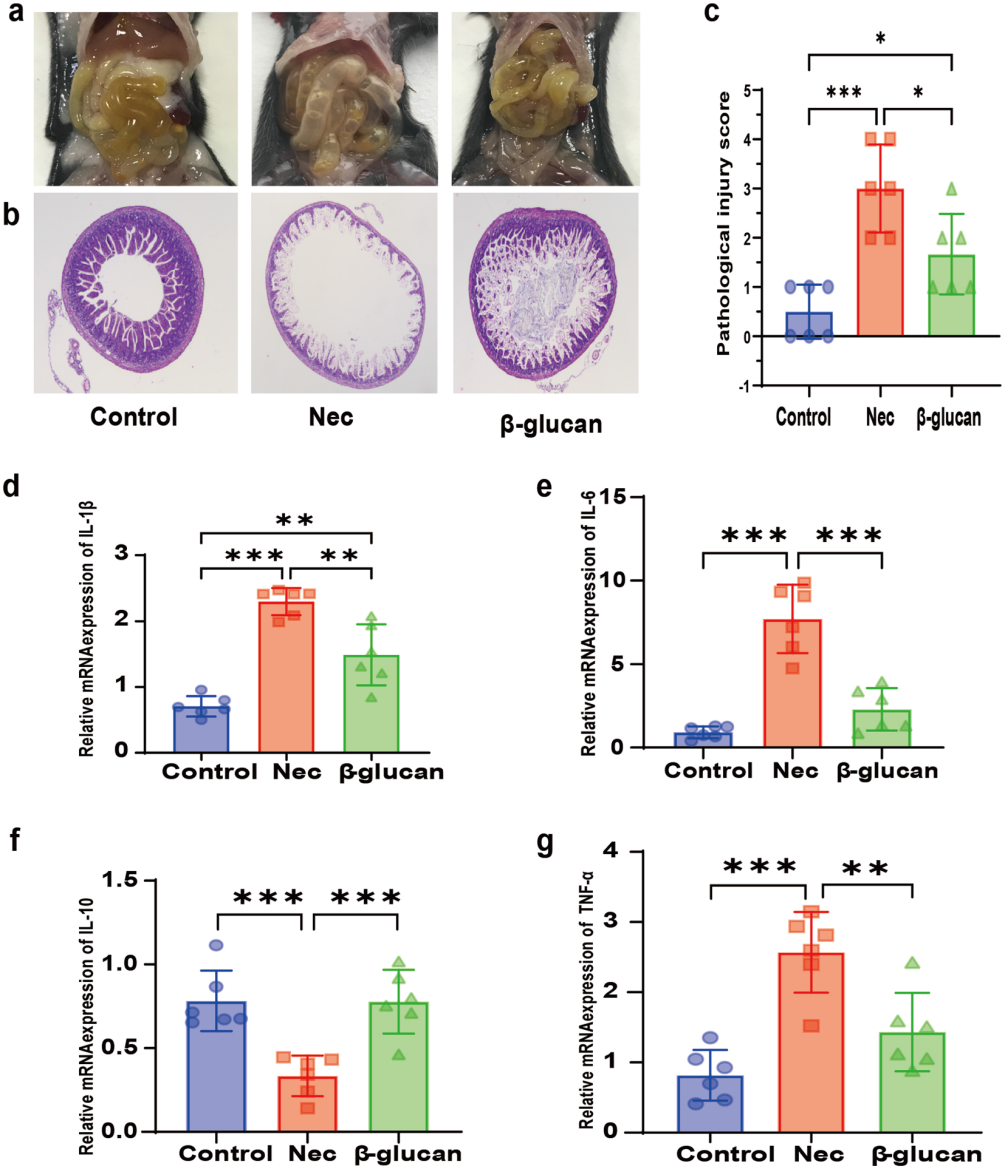
Effects of β-glucan on gross morphology, pathological morphology, histological scoring of intestinal tissue, and the expression of inflammatory cytokines. **a** Gross morphology of the intestine tissues of neonatal mice in the three groups. **b** Images of hematoxylin and eosin staining under light microscopy. Magnification × 40. **c** Comparison of gut histopathological injury scores among the three groups. Control (*n* = 6), NEC (*n* = 6), and β-glucan (*n* = 6). **d**–**g** The relative mRNA expression levels of IL-1β, IL-6, IL-10, TNF-α, *n* = 6–8 in each group *P < 0.05, **P < 0.01, ***P < 0.001

No obvious damage was observed in the Control group (Fig. 2a) but there was gas accumulation and droplet-like changes in the intestinal tissue in the NEC group (Fig. 2a). Only slight gas accumulation and edema were observed in the β-glucan group (Fig. 2a). Based on the observation under a light microscope, a complete intestinal tissue structure, with orderly arranged villi without edema and patchy necrosis, and a thick muscle layer without separation from the lamina propria, was observed in the Control group (Fig. 2b). In the NEC group, the villi were disrupted, patchy necrotic, exfoliated, or even disappeared, and the muscle layer was thin or even fractured (Fig. 2b). In the β-glucan group, the villi were relatively complete with slight edema, and the muscle layer was thicker than in the NEC group. No obvious necrosis or shedding of villi was observed (Fig. 2b). The median intestinal histological pathological score showed that there were statistically significant differences among the three groups (*P* < 0.001), with score in the β-glucan group being significantly lower than that in the NEC group (Fig. 2c,  P < 0.05).

### β-glucan alleviated the inflammatory reaction in intestinal tissue

#### Effects of β-glucan on the productions of proinflammatory cytokines

To investigate the effect of β-glucan on inflammatory cytokines in the intestines of NEC, RT-qPCR was employed to detect the mRNA expression of these cytokines. Compared with the Control group, NEC triggered significantly increased mRNA expression of IL-1β, IL-6, and TNF-α (Fig. 2d, e, g, P  < 0.001). Conversely, β-glucan supplementation remarkably reversed this tendency. β-glucan treatment significantly decreased mRNA expressions of inflammatory factors, such as IL-1β (Fig. 2d,  P< 0.01), IL-6(Fig. 2e,  P< 0.001), and TNF-α (Fig. 2g, P< 0.01), while promoting IL-10 expression (Fig. 2f,  P< 0.001).

#### Effects of β-glucan on TLR4 and NF-κB

**Fig. 3:**
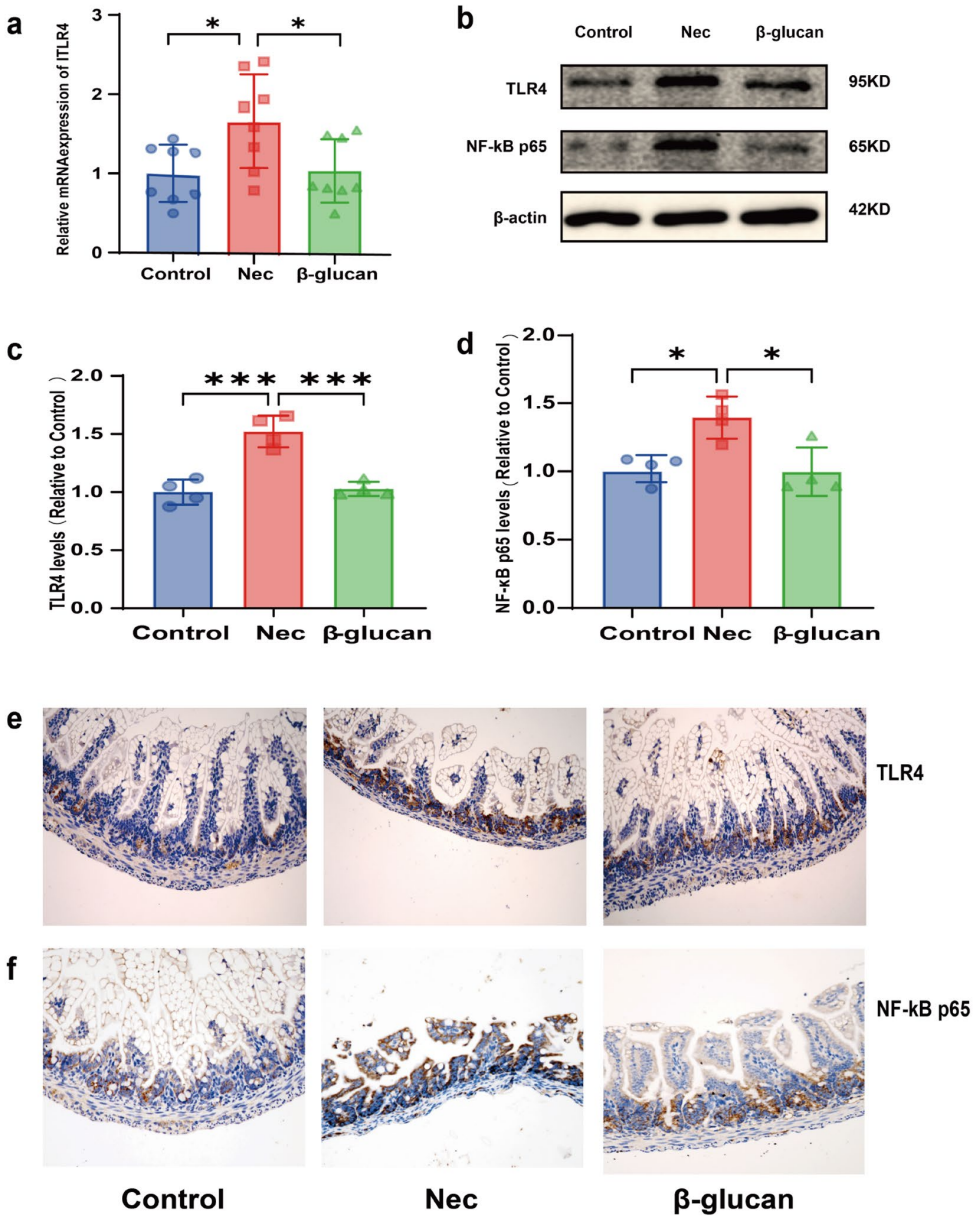
Effects of β-glucan to the TLR4-NF-B pathway in mice with NEC. **a** The relative mRNA expression levels of TLR4. *n* = 6–8 in each group. **b**–**d** Protein expression of TLR4 and NF-B in the three groups determined using western blot. *n* = 4. **e**, **f** TLR4 and NF-B were assessed in the intestinal tissues of the three groups by immunohistochemical staining. Magnification × 200. Data are expressed as the mean ± SD, *P < 0.05, ***P < 0.001

TLR4, widely expressed in intestinal epithelial cells and lymphocytes, plays a key role in the pathogenesis of NEC [10]. When activated by pathogenic microorganisms, TLR4 triggers the innate immune response and subsequently the downstream NF-κB signaling pathway, which ultimately mediates the expression of proinflammatory factors IL-1β, IL-6, and TNF-α [43]. To determine the impact of β-glucan on TLR4-NF-κB intestinal signaling pathway in NEC mice, we assessed TLR4 mRNA expression using real-time qPCR and TLR4 and NF-κB protein expression using western blot and immunohistochemistry. The mRNA and protein expression levels of TLR4 (Fig. 3a,  P< 0.05, Fig. 3b, c, P < 0.001) and the protein expression of NF-κB (Fig. 3b, d, P < 0.05) were increased in the NEC group. However, TLR4 (Fig. 3b, c, P < 0.001) and NF-κB (Fig. 3b, d, P < 0.05) expression was decreased in the β-glucan group.In addition, immunohistochemistry showed much more protein expression of TLR4 (Fig. 3e) and NF-κB (Fig. 3f) compared with the Control and β-glucan group, as demonstrated by enhanced TLR4 and NF-κB immunoreactivity. These results suggested that the amplification of a series of inflammatory cascades involving IL-1β, IL-6 and TNF-α might be suppressed by β-glucan treatment through TLR4-NF-κB pathway, thereby alleviating intestinal inflammation.

**Fig. 4:**
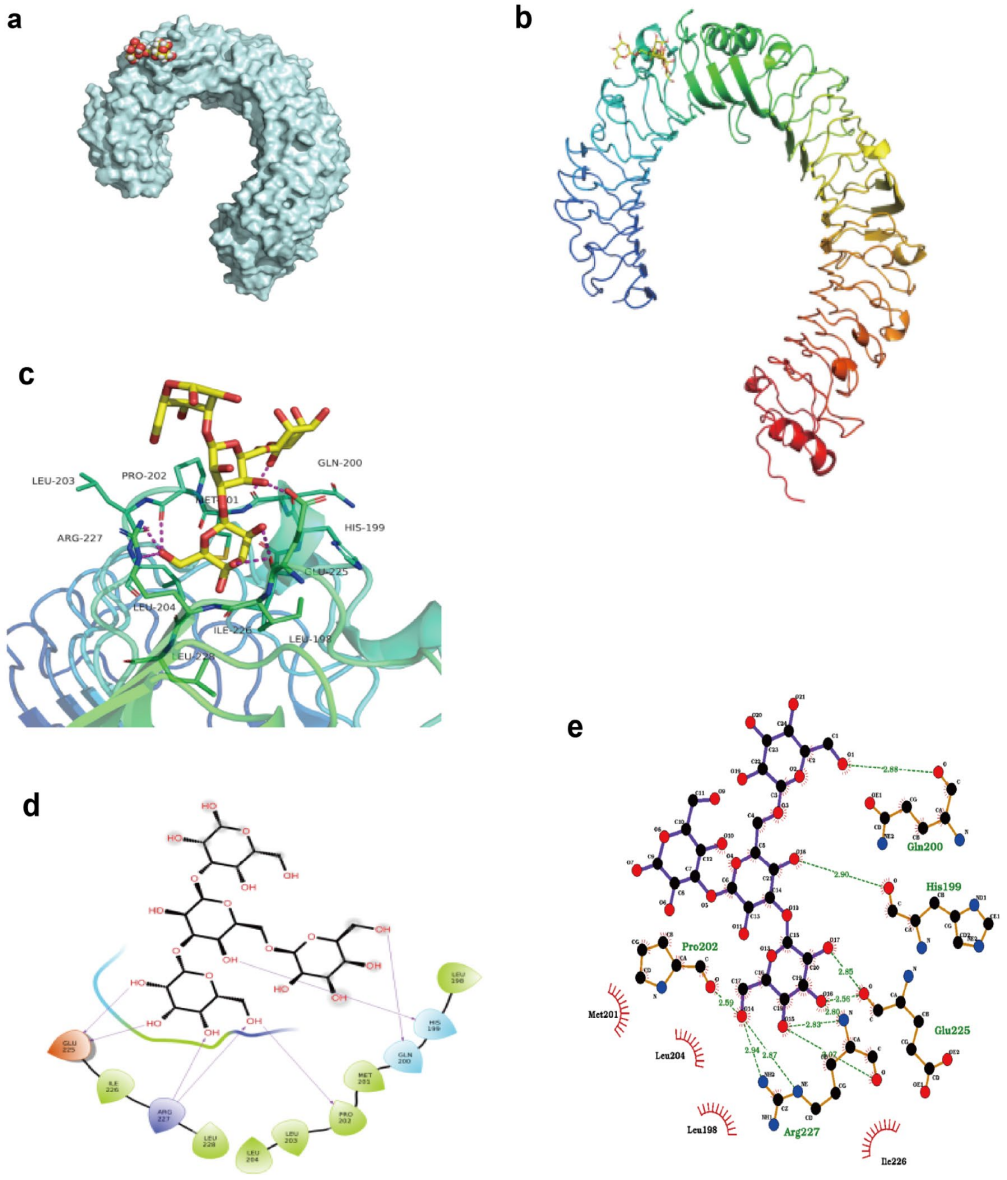
The binding mode between the β-glucan and the TLR4 using molecular docking. **a**–**c** Molecular docking simulation of β-glucan into the active site of TLR4. **d** The results of the binding mode analysis. The meanings of colors in specific amino acids binding with TLR4 are as follows: red stands for negatively charged acidic amino acids, dark blue stands for positively charged basic amino acids, light blue stands for polar amino acids, and green stands for hydrophobic amino acids. **e** The results of LigPlus analysis of hydrogen bonds and hydrophobic interactions. The green dashed line indicates hydrogen bonds, and the red eyelashes indicate hydrophobic interactions, which are directional

To further demonstrate that β-glucan interacted with the active site of TLR4, molecular docking was carried out. As shown in Fig. 4a–e, β-glucan bound to TLR4 mainly through hydrogen bonds and hydrophobic interactions, forming a total of 7 hydrogen bonds whose hydrogen atoms came from the hydroxyl groups of glycoside, and oxygen atoms came from the skeletal carbonyl groups or side chains of the amino acid in TLR4. β-glucan formed 7 hydrogen bonds with the amino acids GLN, HIS 199, GLU 225, ARG 227, PRO 202 of TLR4. β-glucan also formed hydrophobic effect with amino acids ILE 226, LEU 228, LEU 204, LEU203, PRO 202, MET 201, and LEU 198. The best configuration was selected as the most likely binding mode from 30 conformations, with a score of − 60.77 kcal/mol. The highest value of binding energy was estimated at − 19.76 kcal/mol (*Ki* value 3.25fM), indicating that β-glucan displayed a relatively strong binding ability to TLR4. These results suggested that β-glucan exhibited significant affinities to the binding sites of TLR4, subsequently impacting the TLR4-NF-κB signaling pathway, which was consistent with the results in vivo.

### β-glucan improved intestinal barrier function

**Fig. 5:**
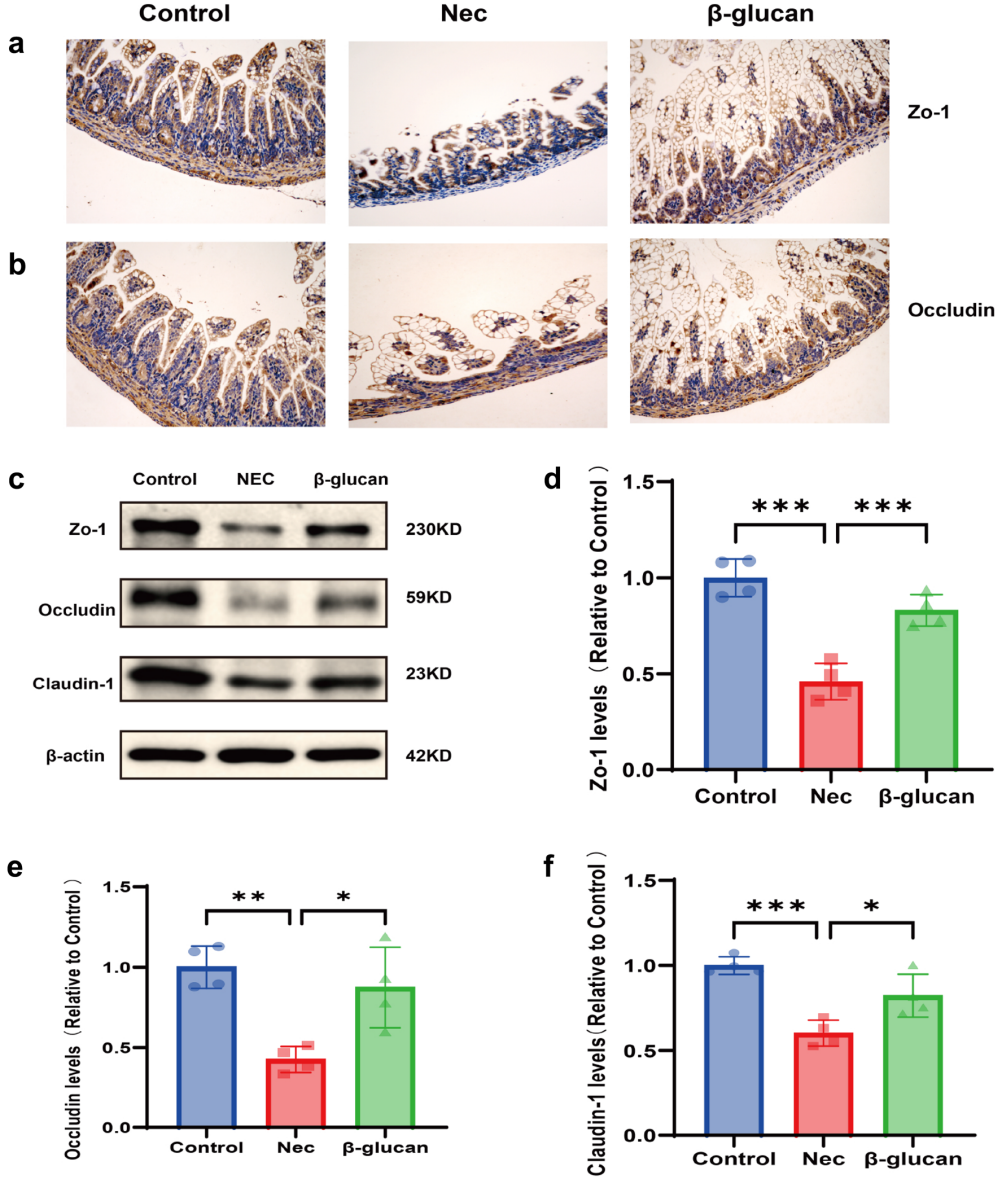
Effects of β-glucan to the tight function proteins in mice with NEC. **a**, **b** The expression of ZO-1 and Occludin was determined in the intestinal tissues of the three groups by immunohistochemical staining. Magnification: × 200. **c**–**f** Protein expression of the ZO-1, Occludin, and Claudin-1 was identified in the intestinal tissues of the three groups using western blot. *n* = 4. Data are expressed as the mean ± SD. *P < 0.05, **P < 0.01, ***P < 0.001

To identify the effect of β-glucan on intestinal barrier integrity, expression of tight junction (TJ) proteins such as ZO-1, Occludin, and Claudin-1 were analyzed using immunohistochemistry and western blot. Immunohistochemistry staining showed much less expression of ZO-1 (Fig. 5a) as well as Occludin (Fig. 5b) in the NEC group compared with the Control and β-glucan group. Western blot showed that the protein expression levels of ZO-1 (Fig. 5c, d, P < 0.001), Occludin (Fig. 5c, e, P < 0.01) and Claudin-1(Fig. 5c, f, P < 0.001) were reduced in the NEC group. By contrast, ZO-1(Fig. 5c, d,  P< 0.001), Occludin (Fig. 5c, e,  P< 0.05), and Claudin-1 (Fig. 5c, f,  P< 0.05) expression were increased in the β-glucan group. These results suggested that β-glucan might prevent NEC-induced disruption of intestinal integrity by enhancing the expression of TJ proteins.

### β-glucan partially changed the gut microbiota in NEC

**Fig. 6:**
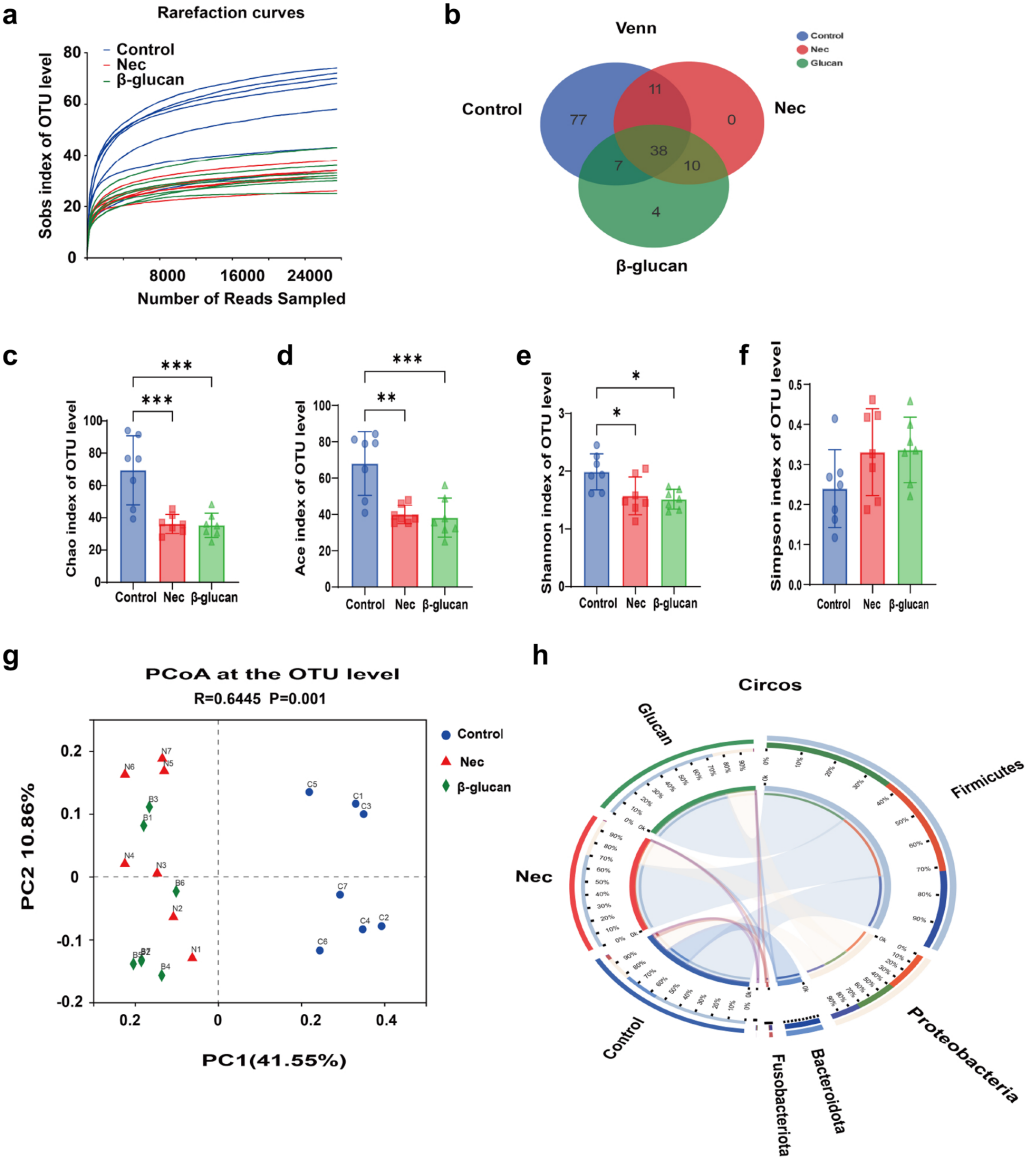
Effects of β-glucan on the gut microbiota in NEC mice. **a** The sparse curve is based on the OTU level of the bacterial community. **b** Overlapping and shared OTU levels in the three groups suggested by the Venn plot. **c**–**f** The α-diversity of gut microbiota was determined using Chao, Ace, Shannon, and Simpson indices. Data are expressed as the mean ± SD. *n* = 7. **g** Unweighted UniFrac-based principal coordinate analysis (PCoA) based on the OTU levels. *n* = 7. **h** Circos plots graphically shows the proportion of each species in each group at the phylum level

**Fig. 7:**
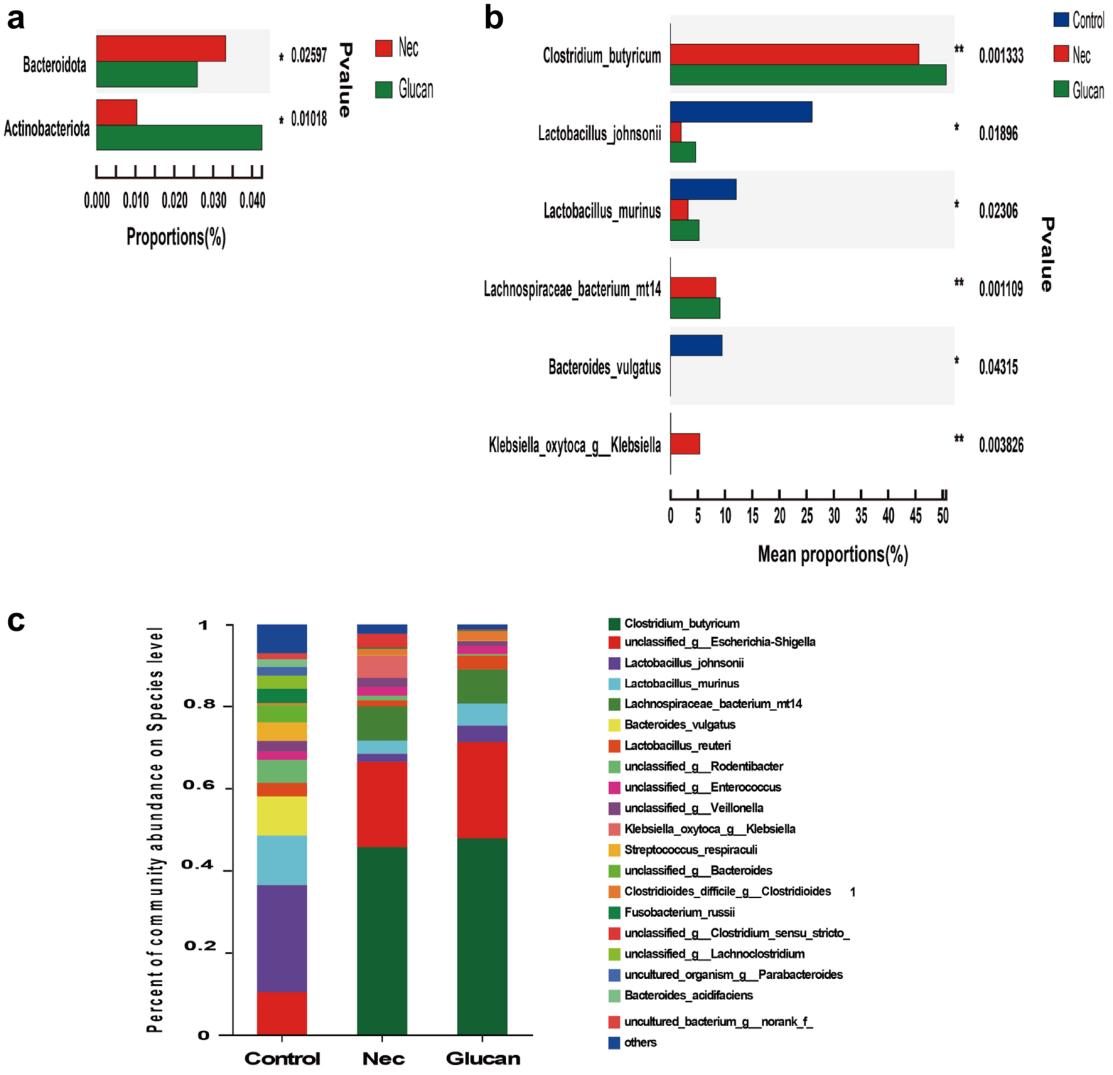
Effects of -glucan supplementation on the gut microbial composition and structure at different taxonomical levels. **a** The flora composition of mice in the β-glucan group and NEC group at the phyla level. **b** The flora composition of mice in the three groups at the species level. **c** The flora composition of mice in the three groups at the genera level

**Fig. 8:**
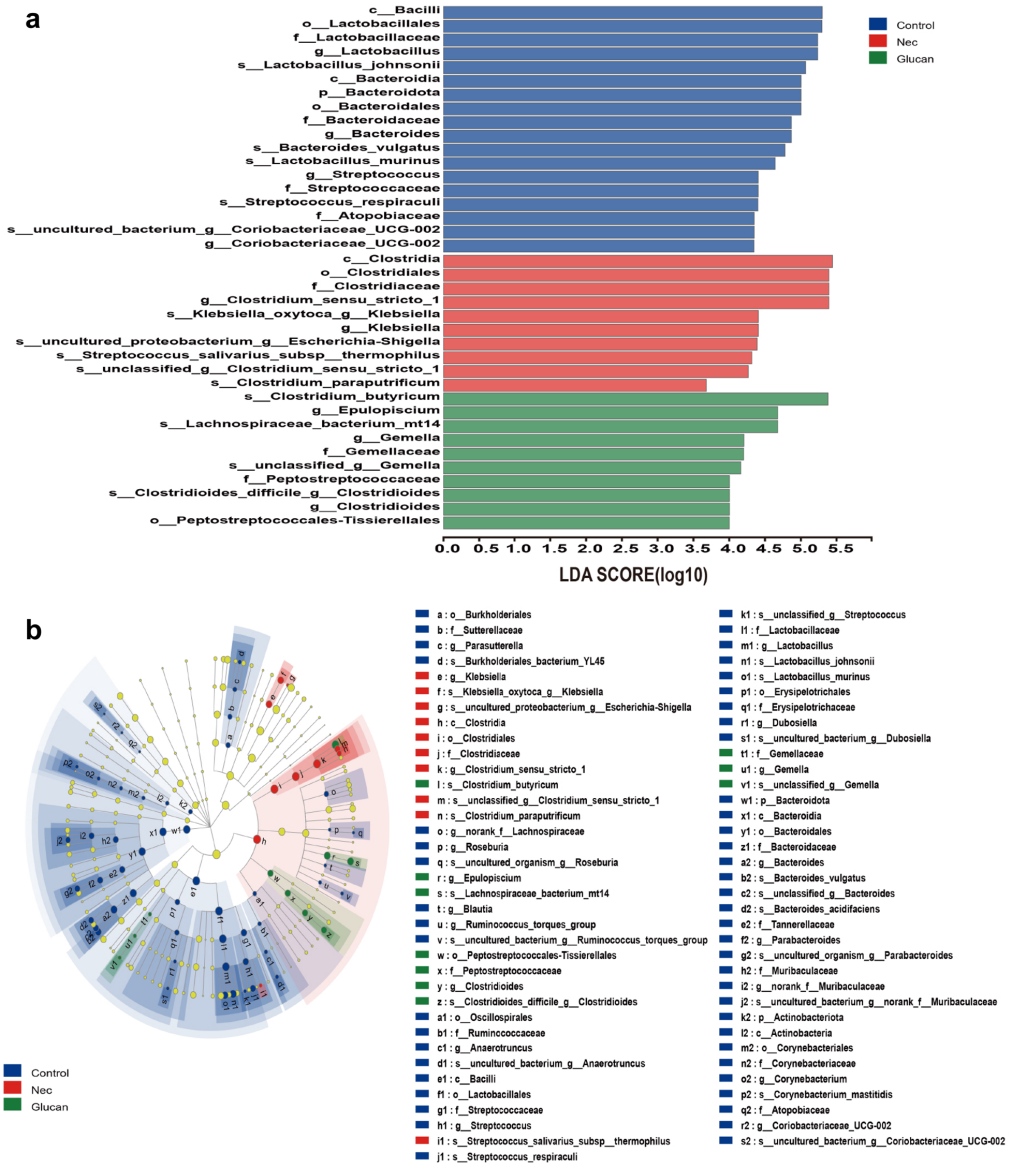
**a** Linear discriminant analysis (LDA) score based on LEfSe analysis showed that the gut microbiota were significantly upregulated by the corresponding treatments. **b** LDA effect size (LEfSe) analysis showed the microbial features most likely to explain the differences between classes

To understand the influence of β-glucan on the gut microbiota in NEC mice, 16S rRNA sequencing was performed. The sparse curve based on the OTU level of the bacterial community gradually reached a saturation plateau along with the increase of sampling readings, indicating that the sequencing depth was sufficient to represent most microbial species (Fig. 6a). The α-diversity of gut microbiota, including Chao1, Ace, Shannon, and Simpson indices, did not differ significantly between the NEC and β-glucan group (Fig. 6c–f), suggesting that β-glucan could not affect the overall bacterial richness and community diversity of mice with NEC. However, PCoA showed that the β-glucan and NEC groups differed from the Control group at the OTU level, but there was some overlap between the NEC and β-glucan groups (Fig. 6g). Venn plots reflected that the β-glucan and NEC groups, respectively, shared 7 and 11 OTUs with the Control group (Fig. 6b). Circos presented that the NEC group was mainly composed of *Firmicutes* (32%) and *Proteobacteria* (40%), whereas the β-glucan group consisted of *Firmicutes* (37%), *Proteobacteria* (32%) and *Bacteroides* (about 0.072%) (Fig. 6h, Additional file 2: Fig. S2). In terms of phyla, the average relative abundance of *Actinomycetes* was lower in the NEC group than in the β-glucan group (Fig. 7a). In terms of genera (Fig. 7b), the relative abundance of *Klebsiella oxytoca g Klebsiella* was obviously higher in the NEC group than in the Control and β-glucan group. The abundance of *Clostridium butyricum, Lactobacillus johnsonii*, *Lactobacillus murinus*, and *Lachnospiraceae bacterium mt14* tended to increase in the β-glucan group compared with the NEC group. In addition, LEfSe analysis is shown in Fig. 8b, and the LDA score based on LEfSe analysis is shown in Fig. 8a.

## Discussion

Numerous studies have suggested that the pathogenesis of NEC is multifactorial, but the etiology remains unclear. An immoderate inflammatory response, dysbiosis of gut microbiota, and bacterial translocation are reported as important in NEC pathogenesis [4]. Our study indicated that early intervention with β-glucan could help alleviate intestinal inflammation, promote gut barrier function, and partially correct the dysbiosis of gut microbiota in a mouse model with NEC.

The incidence of NEC continues to increase with improvements in early neonatal survival, but the mortality rate has not changed due to limitations in prevention and treatment. Although human milk has been reported as an effective preventive measure against NEC [44], most premature babies do not obtain enough milk. Therefore, it is of great significance to explore other effective prevention methods and treatment for NEC.

β-glucan possesses various biological activities mentioned above. Previous studies have demonstrated that oat and *Lentinus edodes* β-glucan can suppress DSS-induced colitis as well as the expression of pro-inflammatory factors in colonic tissues [26, 37]. *Lentinus edodes* β-glucan was identified to show an anti-inflammatory effect by impacting the MAPK-Elk-1 and MAPK-PPARγ signaling pathways and subsequently inhibiting NF-κB activation in in vitro and in vivo studies. NF-κB is one of the most important transcript factors controlling the expression of inflammatory cytokines in which most of inflammatory cytokine genes exist at NF-κB binding sites in the promoter area. It is well known that the TLR4-NF-κB signaling pathway plays a very critical role in the development of NEC [45]. It has been shown that TLR4 expression in intestinal epithelial cells is increased in response to intestinal inflammation in humans and mice [4, 5, 8]. TLR4 activation induces nuclear translocation of NF-κB, which ultimately promotes excessive expression of proinflammatory cytokines, leading to the development of NEC [45]. The proinflammatory cytokines TNF-α, IL-1β, and IL-6 were elevated in infants and animal models with NEC [46, 47], which can be suppressed by the use of TLR4 inhibitors [8, 48]. Our experiment showed that early supplementation with β-glucan reduced TLR4 and NF-κB protein expression levels in NEC mice. In addition, we found β-glucan also effectively inhibited the expression of pro-inflammatory cytokines TNF-α, IL-6, and IL-1β, which is in accordance with the results reported by Minmin Hul [49]. A previous study has also demonstrated that yeast-derived β-glucan could reduce intestinal injury in rat model of NEC by affecting intestinal expression of IL-18, TNF-α, iNOS, CXCL1, Tff3, and Muc-2, which suggests that β-glucan might possess potential in the treatment of NEC via reduction of inflammatory response within the intestine [33]. Moreover, the results of molecular docking also showed that β-glucan had a high affinity for the TLR4 binding site. From the above evidence, we speculate that β-glucan might alleviate intestinal inflammation through the TLR4-NF-κB signaling pathway. However, the present results may not be sufficient to support that β-glucan suppresses the inflammatory cascade amplification induced by NEC. Knockout experiments and determination of more inflammatory factors would be required to prove this hypothesis in the future.

Intestinal barrier dysfunction mainly refers to an abnormal increase in permeability, allowing pathogens to cross the barrier [32], which is regulated by TJs formed between intestinal epithelial cells in the apical region. Functional TJs are essential for maintaining intestinal permeability and intestinal barrier function [50]. Transmembrane proteins Occludin, Claudins, and ZO-1 are reported to be critical for regulating intestinal permeability [51]. Gut barrier injury is suggested to play a key role in NEC pathogenesis [52]. Gut barrier injury leads to an increase in permeability, which subsequently facilitates the translocation of pathogens into the underlying vascular circulation [53]. Meanwhile, elevated proinflammatory cytokines (e.g., TNF-α, IL-1β, and TNF-α) can also disrupt TJs, thus forming a vicious cycle of inflammation and intestinal barrier impairment, leading to further deterioration of gut barrier function and aggravating intestinal inflammation [54]. Hence, restoration of the gut barrier function may contribute to the alleviation of NEC. A previous study has indicated that oat β-glucan attenuated barrier function disruption in DSS-induced colitis mice. Similarly, in this experiment, the down-regulated protein expressions of Occludin, Claudin-1, and ZO-1 in NEC mice were significantly inhibited by β-glucan. These results suggested that the preventive effect of β-glucan against NEC might be tightly related to the improvement of the epithelial TJs.

Gut microbiota is an important part of the intestinal barrier, which is essential for the maturation of intestinal mucosal barrier function, immune system development, nutrient absorption and energy metabolism [55, 56]. Numerous intestinal bacteria combine with intestinal epithelial cells to construct a strong barrier, which have the ability to resist the invasion of pathogens colonized in the intestinal tracts [57]. However, various prenatal or postnatal risk factors lead to a decreased diversity in gut microbiota, which disturbs the structure of gut microbiota and leads to a weak capacity of the intestinal barrier against potential pathogens in infants with NEC [58, 59]. In addition, the metabolites released by pathogenic bacteria contribute to the destruction of the gut barrier and invasion by bacteria in the intestines, inducing an intestinal inflammation that finally leads to the occurrence of NEC [60, 61]. For example, Gram-negative bacteria in the immature intestines of preterm infants can produce LPS to activate the TLR4 signal pathway [62], mediate inflammatory response, trigger the secretion of various proinflammatory factors, and ultimately facilitate the progression of NEC [63, 64]. By contrast, beneficial bacteria have the ability to produce metabolites, such as the butyric acid, which enhances the gut barrier, regulates the activation of NF-κB pathway, reduce the produce of pro-inflammatory factor, inhibit intestinal inflammation [65, 66]. Therefore, modulating gut microbiota may provide new insights into therapeutic strategies for infants with NEC. In this study, we found that β-glucan did not change the diversity of intestinal microbiota in the NEC mice at the OTU level. The possible reason is that the β-glucan used in this study is from fungi, whose ability to modulate the intestinal microbiota is not as good as β-glucan from plants. In terms of phyla, *Firmicutes* and *Proteobacteria* showed no significant differences among the three groups, but the relative abundance of *Actinobacteria* in the β-glucan group was higher than that in the NEC group. Early studies have revealed that microbial dysbiosis before NEC in preterm infants is characterized by an increased relative abundance of *Proteobacteria* and decreased abundance of *Firmicutes*, *Bacteroidetes*, and *Actinobacteria* [67]. Although our study showed that β-glucan could not significantly decrease the abundance of *Proteobacteria* and increase the abundance of *Bacteroidetes* and *Firmicutes*, its pretreatment increased the abundance of *Actinobacteria*. Moreover, the abundance of *Clostridium butyricum*, *Lactobacillus johnsonii*, *Lactobacillus murinus* and *Lachnospiraceae bacterium mt14* increased in response to β-glucan administration. Previous studies have found that after birth, the newborn gut is gradually colonized with facultatively and strictly anaerobic bacteria, including *Clostridium butyricum* [68]. *Clostridium butyricum* is a species that has a variety of known strains, including toxigenic and nontoxigenic strains [69, 70]. Cytotoxic *Clostridium butyricum* strains have been demonstrated to be associated with the occurrence of NEC [71]; however, nontoxigenic strains of *Clostridium butyricum* have been used as probiotics for decades in a wide range of human diseases, including irritable bowel syndrome, inflammatory bowel disease, neurodegenerative disease, and metabolic disease [68, 72]. Hayashi et al. demonstrated that *Clostridium butyricum* supplementation can prevent DSS-induced colitis in mice by promoting IL-10 production by intestinal macrophages in inflamed mucosa [73]. *Lactobacillus johnsonii* and *Lactobacillus_murinus* are both species in the genus *Lactobacillus*. Recent studies have shown a lower relative abundance of *Lactobacillus* in NEC cases or before NEC onset [74–76]. Mubina et al. pointed out that the probiotic *Lactobacillus murinus* significantly protected rats against NEC by colonizing the neonatal intestine and reducing the gut barrier damage [77]. *Lachnospiraceae* is a protective commensal strain that produces short-chain fatty acids such as butyrate by fermentation of dietary fiber [78]. Recent research by He et al. demonstrated that the administration of butyrate could reduce intestinal inflammation in NEC mice possibly via the induction of T_reg_ [79]. We also found that β-glucan pretreatment can reduce the abundance of *Klebsiella oxytoca g Klebsiella* compared with the NEC group. *Klebsiella oxytoca* is a recently emerging pathogen that may cause the outbreak of NEC [80]. To sum up, we speculate that β-glucan could partially modulate the gut microbiota by increasing the diversity of beneficial bacteria whereas reducing the relative abundance of pathogenic bacteria. The increased diversity in beneficial bacteria as well as their metabolites subsequently might regulate the inflammatory cells and protect the intestine against inflammation induced by NEC. However, future experiment of fecal microbial transplantation using germ-free mice will be needed to explore the exact mechanisms concerning the effects of β-glucan from the aspect of the gut microbiota.

## Conclusions

**Fig. 9:**
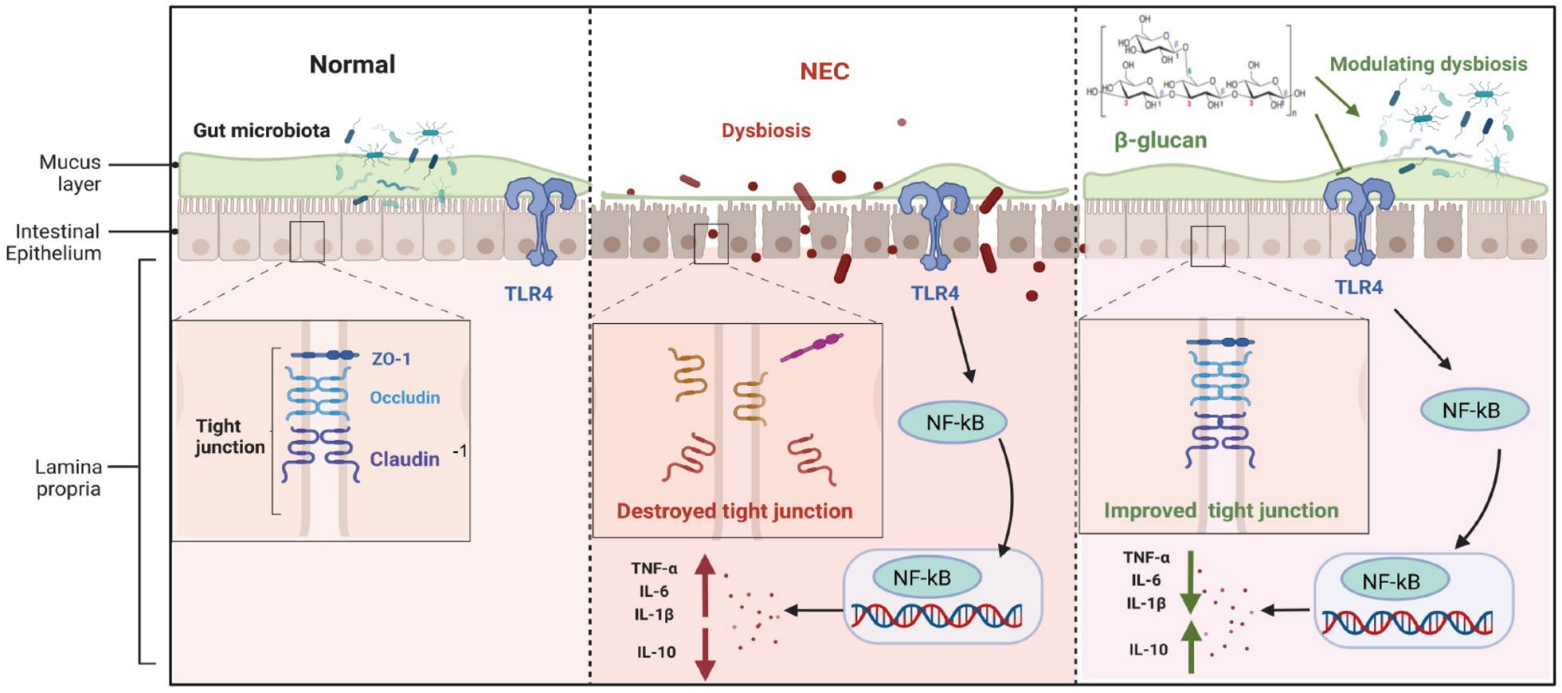
Summary scheme of the mechanisms underlying the preventive effect of β-glucan on necrotizing enterocolitis. Necrotizing enterocolitis is characterized by disturbed gut microbiota, destroyed gut barrier, activated TLR4, and excessive inflammatory cytokine levels. -glucan suppressed the TLR4-NF-B signaling pathway, and thus decreased inflammatory cytokine levels induced by necrotizing enterocolitis. β-glucan protected intestinal epithelial barrier disruption induced by necrotizing enterocolitis, via up-regulating the tight junction protein expressions, including ZO-1, Occludin, and Claudin-1

In conclusion, important findings of this current study were that β-glucan might intervention alleviated intestinal inflammation through the TLR4-NF-κB pathway, improved gut barrier, and partially modulated gut microbiota, ultimately protected against NEC in a mouse model. Summary scheme of the mechanisms underlying the preventive effect of β-glucan on NEC was shown in Fig. 9. However, this experiment is based on an animal study and the exact mechanism and safety of β-glucan need to be completely elucidated in further cellular studies. In addition, due to the limited sample size, the dose of β-glucan was selected according to the results of pre-experiments. Further research is needed to explore whether different concentrations of β-glucan have different preventive effects against NEC in cell experiments.

## Supplementary Information

**Additional file 1. Table S1. **Primers used for Quantitative Real-Time PCR.

**Additional file 2.**. Circos plot presented the proportion of *Bacteroides* in the three groups.

## Data Availability

All data generated or analysed during this study are included in this published article.

## Abbreviations

NEC: Necrotizing enterocolitis
TLR4: Toll-like receptor 4
NF-κB: Nuclear factor-κB
LPS: Lipopolysaccharide
TNF-α: Tumor necrosis factor-α
IL-6/1β/10: Interleukin-6/1β/10
PBS: Phosphate buffer saline
HRP: Horseradish Peroxidase
DAB: 3,3’-Diaminobenzidine
RIPA: Radio immunoprecipitation assay lysis buffer
PVDF: Polyvinylidene difluoride
ECL: Enhanced chemiluminescence
OTU: Operational taxonomic units
