# Supplementary material for: β-glucan protects against necrotizing enterocolitis in mice by inhibiting intestinal inflammation, improving the gut barrier, and modulating gut microbiota
Source: J Transl Med. 2023 Jan 10;21:14. doi: 10.1186/s12967-022-03866-x (PMC9830848; doi:10.1186/s12967-022-03866-x)
Supplement: Supplementary file 1 — Additional file 1. Table S1. Primers used for Quantitative Real-Time PCR. [file 12967_2022_3866_MOESM1_ESM.docx]

**Table S1.** Primers used for Quantitative Real-Time PCR

| **Mouse Gene** | **Forward Primer (5’**→**3’)** | **Reverse Primer (5’**→**3’)** |
| --- | --- | --- |
| IL-1β | GTCGCTCAGGGTCACAAGAA | AGAGGCAAGGAGGAAAACACA |
| IL-6 | CTTCTTGGGACTGATGCTGGT | GGTCTGTTGGGAGTGGTATCC |
| IL-10 | TGGACAACATACTGCTAACCGA | AATGCTCCTTGATTTCTGGGC |
| TNF-α | CCTTGTTGCCTCCTCTTTTGC | GTAGGGCGATTACAGTCACGG |
| TLR-4 | CTGGGGCTCATTCACTCACTA | CTCAGACTCGGCACTTAGCA |
| β-actin | TGAGAGGGAAATCGTGCGTGAC | GCTCGTTGCCAATAGTGATGACC |
