# Supplementary figures and images for: β-glucan protects against necrotizing enterocolitis in mice by inhibiting intestinal inflammation, improving the gut barrier, and modulating gut microbiota
Source: J Transl Med. 2023 Jan 10;21:14. doi: 10.1186/s12967-022-03866-x (PMC9830848; doi:10.1186/s12967-022-03866-x)

Fig. S2 Circos plot presented the proportion of *Bacteroides* in the three groups.

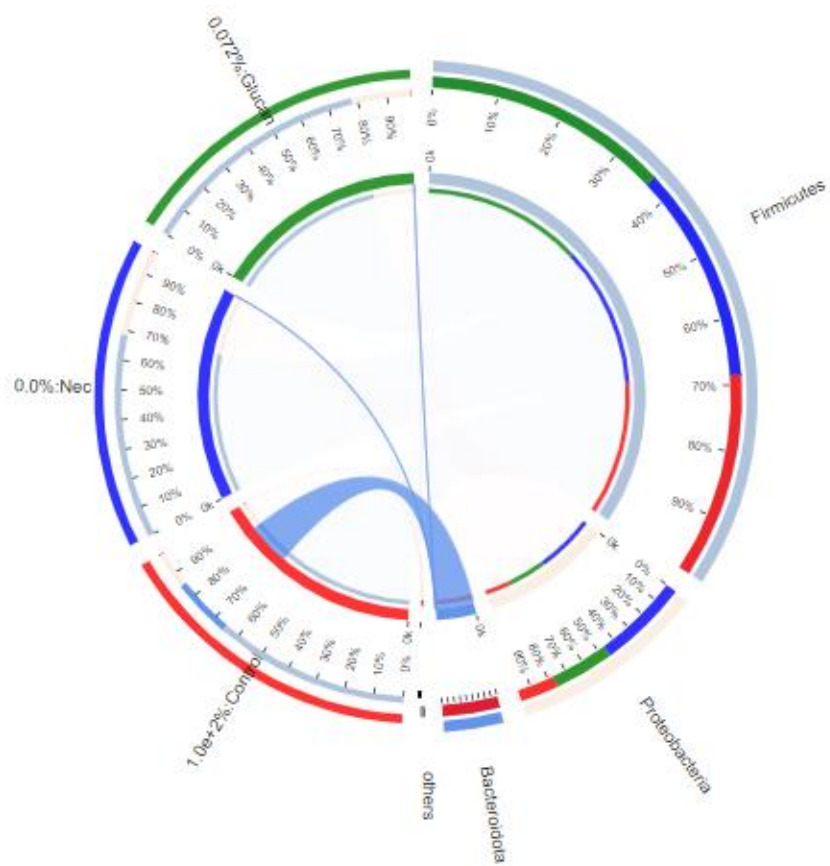

Supplement: Supplementary file 2 — Additional file 2.. Circos plot presented the proportion of Bacteroides in the three groups. [file 12967_2022_3866_MOESM2_ESM.pdf]
